# Medical Society of the County of Erie

**Published:** 1899-03

**Authors:** F. C. Gram

**Affiliations:** Secretary


### Society Proceedings.

### MEDICAL SOCIETY OF THE COUNTY OF ERIE.

Reported by F. C. Gram, M. D., Secretary.

A SPECIAL meeting of the Medical Society of the County of Erie was held at the Fitch Institute, at 10 a. m., January 23, 1899.

The President, Dr. J. B. Coakley, stated that the purpose of the meeting was to take official cognisance of the death of Dr. A. H. Crawford.

The following memorial was then presented by Dr. U. C. Lynde:

Whereas, Dr. Albert H. Crawford, after a life of professional usefulness, has met the universal fate of mankind; and

Whereas, In his long membership in the Erie County Medical Society he never, intentionally, violated its laws, either in his relation to the Society or its individual members; and

Whereas, He was ever careful and watchful of the best interests of his patients; and

Whereas, When sick, weak and emaciated, when he must have known the sands of life were about exhausted, he like a brave soldier in the line of his duty, heroically continued his labor until at last he fell mortally wounded; therefore

*Resolved*, That this Society in the death of Dr. Albert H. Crawford recognises the loss of one of its oldest and most intelligent members.

*Resolved*, That we commend his example to this Society and the profession generally; and that we extend to his wife and children our sympathy on this, the saddest of human events.

*Resolved*, That this Society as a tribute to his memory attend his funeral services in a body.

Remarks were made by the members present, whereupon the memorial was adopted and all proceeded to pay their last respects to the dead.

---

At Aargan, in Baden, some recent excavations have disclosed an ancient Roman military hospital containing remains of all sorts of medical and pharmaceutical appliances, together with numerous crude surgical appliances.—*Medical Age.*

